# Molecular Mechanisms of KDELC2 on Glioblastoma Tumorigenesis and Temozolomide Resistance

**DOI:** 10.3390/biomedicines8090339

**Published:** 2020-09-10

**Authors:** Yu-Ling Tsai, Hsin-Han Chang, Ying-Chuan Chen, Yu-Chan Chang, Ying Chen, Wen-Chiuan Tsai

**Affiliations:** 1 Department of Pathology, Tri-Service General Hospital, National Defense Medical Center, Taipei 114, Taiwan; c909228@gmail.com; 2 Graduate Institute of Life Science, National Defense Medical Center, Taipei 114, Taiwan; albertchang1008@gmail.com; 3 Department of Biology and Anatomy, National Defense Medical Center, Taipei 114, Taiwan; ychen0523@mail.ndmctsgh.edu.tw; 4 Department of Physiology and Biophysics, National Defense Medical Center, Taipei 114, Taiwan; addy0918@gmail.com; 5 Department of Biomedical Imaging and Radiological Sciences, National Yang-Ming University, Taipei 115, Taiwan; jameskobe0@gmail.com; 6 Graduate Institute of Pathology and Parasitology, National Defense Medical Center, Taipei 114, Taiwan

**Keywords:** KDELC2, glioblastoma, Notch, temozolomide

## Abstract

The activation of the Notch pathway induces glioblastoma (GBM) development. Since KDEL (Lys-Asp-Glu-Leu) containing 2 (KDELC2) is involved in the Notch pathway, the detailed mechanism is still undetermined. The Cancer Genome Atlas (TCGA) and Chinese Glioma Genome Atlas (CGGA) databases revealed that KDELC2 mRNA was associated with oncologic factors of GBM. U87, LN229, LNZ308, U118MG, and GBM8401 cells showed higher KDELC2 expression than normal brain tissues. The results of MTT, wound healing, and invasion assays proved that KDELC2 knockdown suppressed GBM-aggressive behaviors. The inhibitory properties of GBM stemness and angiogenesis under KDELC2 knockdown were evaluated by tumor spheroid and tube formation assays. Suppression of KDELC2 downregulated Notch factors’ expressions, including KDELC1, pofut1, Notch receptors 1–3, and HES-1. Immunoblot assay showed that KDELC2 knockdown promoted tumor apoptosis by downregulating PI3k/mTOR/Akt, MAPK/ERK, and NF-kB pathways. The combination of KDELC2 knockdown and temozolomide (TMZ) treatment had an optimal therapeutic effect by suppressing MGMT expression. Results of an orthotopic xenograft animal model and human tissue confirmed that KDELC2 correlated with glioma proliferation, advanced grades, and poor prognosis. Therefore, KDELC2 might be a potential pharmacological target to inhibit tumorigenesis, epithelial–mesenchymal transition, angiogenesis, and chemo-resistance of GBM.

## 1. Introduction

Among all primary brain tumors, GBM has the most aggressive behavior and worst prognosis [1]. The molecular evidence showed the association of various signaling pathways with glioma development, including Notch, phosphatidylinositol-3-kinase (PI3K)/protein kinase B (Akt)/rapamycin (mTOR), and nuclear factor kappa-light-chain-enhancer of activated B cells (NF/kB) signaling pathways [2,3,4]. The switch differentiation of neural crest stem cells from neurogenesis to gliogenesis was initiated by transient Notch activation [5]. Recently, Notch deregulation was found to impact brain cancer stem cell differentiation by Notch intracellular domain (NICD) overexpression [6]. However, Li et al. [7] performed a bio-informative analysis to evaluate the expression of possible genes and signaling pathways in GBM. Compared with normal brain tissue, downregulated KDELC2 expression was identified in GBM patients [7]. To date, the role of KDELC2 in gliomas still needs to be clarified.

Notch receptors are composed of NICD and Notch extracellular domain (NECD). The main part of NECD primarily includes 36 epidermal growth factor (EGF)-like motifs [8,9]. NECD comprises abundant surface-modified *O*-linked glycans, including *O*-glucose (*O*-Glc), *O*-fucose (*O*-Fuc), and *O*-GlcNAc [8,10]. These modifications strengthen the linkage between the Notch receptor and ligands and activate Notch signaling [11]. Initially, Notch receptors enter the endoplasmic reticulum (ER) and Golgi apparatus (Golgi) and are modified by some glycans and glycosyltransferases, including Rumi, Pofut1, *O*-glucosyltransferase 1 (Poglut1), Poglut2 (KDELC1), and Poglut3 (KDELC2) [12]. KDELC1 and KDELC2 aid transfer of *O*-glucose to Notch 1 EGF11 and Notch 3 EGF10 [11]. Recently, the Notch receptor activation relied on Pofut1 and Poglut1 expression [13,14,15,16]. However, the relationship of KDELC2 with Notch receptor molecules remains unknown.

Herein, we successfully demonstrated that KDELC2 expression induced GBM tumorigenesis and aggressive behaviors through the activation of Notch and associated cascade signaling pathways. Therefore, KDELC2 was considered not only a critical factor for GBM development but also a good predictor of chemotherapeutic effect and overall prognosis.

## 2. Materials and Methods

### 2.1. In Silico Study

All clinical parameters and genomic data of TCGA and CGGA databases were obtained from the following website: https://xenabrowser.net/heatmap/. Additionally, we searched the Gene Express Omnibus website to explore series datasets of gene expression by Genspring software (version 13.1.1, Agilent, Santa Clara, CA, USA), as previously described [17]. Furthermore, some data on KDELC2 expression with the World Health Organization (WHO) classification, IDH1 mutation, tumor progression, and 1p/19q co-deletion were also downloaded from the CGGA database.

### 2.2. Normal Brain Lysates, Human Glioma Cell Preparation, and Western Blot Analysis

The human GBM cell lines GBM8401, LN229, U87MG, U118, and T98G were obtained from the laboratory room of Dr. Ying-Chuan Chen, National Defense Medical Center. GBM8401 was maintained in Dulbecco’s modified Eagle’s medium (DMEM) with 5% fetal bovine serum (FBS). LN229, U87MG, and U118MG were maintained in DMEM with 10% FBS. T98G was maintained in F12/DMEM with 10% FBS. Human umbilical vein endothelial cells (HUVECs) were maintained in the extracellular matrix (ECM) containing basic fibroblast growth factor. The cell lysates were prepared from 2 × 10^7^ cells GBM cell lines. Normal human brain lysates were purchased from GeneTex, Irvine, California, USA. Western blot (WB) assay protocol was followed as previously described [18]. All primary antibodies and their detailed information are listed in Supplementary Table S1.

### 2.3. RNA Isolation and Real-Time Reverse Quantitative Transcription-Polymerase Chain Reaction (qRT-PCR) 

Total RNA was extracted (PAXgeneTM Blood RNA kit, PreAnalytix) using the RNase-Free DNase set (Qiagen, Hilden, Germany). We used the ThermoScript real-time RT-PCR system (Invitrogen, Carlsbad, California, USA) to prepare single-stranded cDNA from 1 g of total RNA. Real-time quantitative reverse transcription polymerase chain reaction (qRT-PCR) was performed following the previous protocol [18]. All experimental primers were purchased from Search-LC (Heidelberg, Germany). The detailed sequences of included primers are listed in Supplementary Table S2.

### 2.4. Stable Expression of shRNAs 

Recombinant lentiviruses were produced by co-transfection of HEK293T cells with pCMVdeltaR8.91, pMD.G, and pLKO. 1-puro vectors containing KDELC2-specific shRNA (shKDELC2) or luciferase-specific shRNA (shLuc) were used as controls (National RNAi Core Facility, Academia Sinica, Taipei, Taiwan). GBM8401 or U87 were then infected with lentivirus-bearing shKDELC2 or shLuc respectively, and incubated with puromycin to determine the stably infected cells.

### 2.5. MTT Assay

Both GBM8401 and U87 GBM cells with transfected shKDELC2 or shLuc were seeded at a density of 1.5 × 10^3^ cells per well in 96-well plates. After 24, 48, or 72 h, cell proliferation was determined by the 3-(4,5-dimethylthiazol-2yl)-2,5-diphenyltetrazolium bromide (MTT) assay. Optical density (OD) was detected at 595 nm with a microplate reader. All assays were independently repeated three times.

### 2.6. Wound Healing and Cell Invasion Assays

Initially, we seeded 2 × 10^5^ GBM8401 and U87 GBM cells at a density of 3 × 10^5^ in a 3.5 cm dish overnight. Then, 200 µL tips were used to scratch the above-seeded glioma cells within 48 h after transfection of shLuc, shKDELC2 #180, or shKDELC2 #220. The seeded cells were washed with phosphate-buffered saline (PBS), and the scratched area was photographed under a microscope at 0, 24, and 48 h. Further, a transwell invasion assay was performed using 24-well BD Matrigel Invasion Chambers (BD Biosciences, Franklin Lakes, NJ, USA). Cell suspensions (5 × 10^4^ cells) were seeded into the upper chambers. After 24 h, the invading cells were fixed with methanol, stained with 0.5% crystal violet, and then photographed using a microscope.

### 2.7. Gelatin Zymography

Matrix metalloproteinase-2 (MMP2) activity was determined by gelatin zymography using the conditioned medium. Equal amounts of the conditioned medium (40 μL) were mixed with a sample buffer (1 M Tris-HCl (pH 6.8), 50% glycerol, 10% SDS, and 0.5% bromophenol blue). Then, the samples were loaded onto a 10% SDS-PAGE containing 0.1% gelatin. The gels were washed three times with renaturing buffer (50 mM Tris-Hcl (pH 7.4)) for 45 min and incubated with an incubation buffer at 37 °C for 3 days. Finally, the gel was stained with Coomassie Blue R-250 for 1 h and de-stained with a de-staining buffer (2:1 methanol:acetic acid solution) for 2 h at room temperature until a clear zone was visible.

### 2.8. Flow Cytometry Analysis 

GBM8401 and U87 glioma cells with shLuc or shKDELC2 transfection were seeded at a density of 2 × 10^5^ cells in 6-well plates. The cells were harvested and washed with PBS, and then fixed in 80% ice-cold ethanol overnight. These fixed cells were incubated with PBS containing 10 μg/mL propidium iodide and 0.5 mg/mL RNase A for 15 min at 37 °C. All samples were examined using a FACS Calibur machine (BD Biosciences). 

### 2.9. Immunofluorescence (IF) Staining 

The cells were grown on coverslips, then fixed with 4% paraformaldehyde and permeabilized in 0.1% Triton X-100. The cells were stained with some antibodies (listed in Supplementary Table S3) at 4 °C for 16 h. After washing with PBS, cells were stained by Alexa Fluor 488-conjugated anti-mouse IgG or Alexa Fluor 488-conjugated anti-rabbit IgG antibody for 1 h, and then stained with DAPI for 3 min.

### 2.10. Three-Dimensional (3D) Collagen Spheroid Assay

GBM8401 and U87 cells with shLuc or shKDELC2 transfection were mixed with 5 mg/mL Matrigel matrix (BD Biosciences), seeded into a 24-well plate at a density of 3 × 10^4^ cells, and pre-coated with the Matrigel matrix. After 2 weeks, all invading cells were fixed with 4% paraformaldehyde and then photographed under a microscope.

### 2.11. Tube Formation Assay 

A 50 μL Matrigel matrix (BD Biosciences, Franklin Lakes, NJ, USA) was slowly thawed on ice and added to each of the 96-well plates at 37 °C for 30 min. Then, HUVECs were plated at a density of 1 × 10^4^ on top of the Matrigel matrix and treated with the supernatant from cultured GBM8401 or U87 GBMs for 12 h. After incubation, the number of tubes and nodes of the tubular structures was quantified.

### 2.12. Orthotopic Xenograft Animal Model 

In total, 25 eight-week-old female BALB/c AnN.Cg-*Foxnlnu*/CrlNarl mice were purchased from the National Laboratory Animal Center, Taipei, Taiwan. One week later, 1 × 10^5^ GBM8401-Luc tumor cells were implanted into the right cerebral hemisphere of the mice. The animals were randomly divided into four groups: shLuc, shLuc + Temozolomide (TMZ, MedChem Express, NJ, USA), shKDELC2, and shKDELC2 + TMZ treatment groups. TMZ was administered through oral gavage at 5 mg/kg/day for 7 days. The region of interest (ROI) was monitored using a noninvasive in vivo imaging system (IVIS) (Perkin Elmer, Massachusetts, USA) and measured at 0, 2, 5, and 7 days of TMZ administration. The bioluminescence intensity was compared after intraperitoneal injection of D-luciferin firefly and potassium salt (Biosynth, Thal, Swiss) in PBS. At 7 days, the mice were euthanized with tiletamine-zolazepam and xylazine, and the brains were fixed in 10% formalin, embedded in paraffin, and cut into serial sections. Animal experiments were approved by the Institutional Animal Care and Use Committee of the National Defense Medical Center, Taiwan (Approval number: IACUC-20-106, Date: 14 April 2020).

### 2.13. Tissue Microarray Slide Preparation and Immunohistochemical (IHC) Staining

We prepared two sets of tissue microarrays (no. GL2083a and no. GL2083b) from GenDiscovey Biotechnology Inc. To exclude the incomplete tissue cores and incompatible diagnosis from microscopic picture and datasheet, 77 glioma and 5 non-neoplastic brain tissues were included in this study. According to the 2016 WHO Classification of Tumors of the Central Nervous System, some biomarkers were applied to explore the relationship between KDELC2 expression and some well-known oncogenic factors of human gliomas. Antigen retrieval and immunohistochemical (IHC) staining were performed following the protocol described previously [18]. Detailed information of the concentration and the included biomarkers is listed in Supplementary Table S4.

### 2.14. Assessment of IHC Scores of KDELC2 in Human Glioma Tissues

The IHC scores of KDELC2 were obtained by multiplying the corresponding percentage in each human glioma tissue core. The cytoplasmic staining intensity of tumor cells was scored on a scale of 0 (absence of staining), 1 (weak staining), 2 (moderate staining), or 3 (strong staining). The microscopic magnifications of 40×, 20×, and 10× or 4× were viewed as weak, moderate, and strong staining, respectively. If the tumor cells showed weak staining (less than 5%), the intensity score was negative. Therefore, IHC scores of each tissue sample ranged from 0 to 300. KDELC2 expression in normal endometrial tissue was viewed as positive control.

### 2.15. Statistical Analysis of KDELC2 Expression and Overall Survival Time of Human Glioma Tissues

To evaluate the relationship between KDELC2 IHC expression and overall survival time, a Kaplan–Meier survival test was performed, and a *p* < 0.05 was considered to indicate statistical significance. All included human glioma tissues were divided into two groups based on the average KDELC2 IHC scores to prevent patient number bias in each group. We used the Kaplan–Meier survival method to evaluate KDELC2 expression and overall survival.

## 3. Results

### 3.1. KDELC2 mRNA Expression Correlates with Non-GCIMP and IDH1 Wild-Type GBMs in the TCGA Database

**Figure 1:**
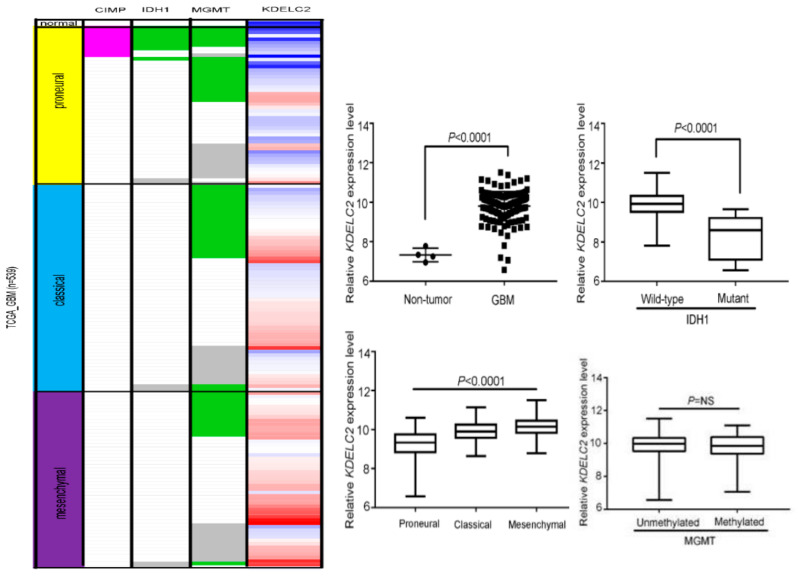
Expression analysis of KDELC2-induced tumorigenesis in glioblastomas (GBMs) in The Cancer Genome Atlas database. KDELC2 mRNA overexpression correlated with mutant IDH1, mesenchymal subtype, G-CIMP, but was not related to MGMT methylation of GBMs. Bars, means ± SEM. * *p* < 0.05; ** *p* < 0.01; *** *p* < 0.0001; *p* = NS, non-significant.

To evaluate the tendency of KDELC2 expression in GBMs, we enrolled 539 patients from the TCGA database in our study. Compared with non-neoplastic brain tissue, KDELC2 overexpression was more commonly identified in GBM patients (Figure 1). KDELC2 mRNA expression was higher in the mesenchymal subtype than in classical and proneural subtypes of GBMs (Figure 1). Additionally, our results from the TCGA database showed significantly higher KDELC2 expression in non-GCIMP than in GCIMP GBMs (Figure 1). Furthermore, KDELC2 expression correlated with isocitrate dehydrogenase 1 (IDH1) wild-type (Figure 1). Unfortunately, the status of methylation of MGMT did not reach statistical correlation with KDELC2 expression in GBM patients.

### 3.2. KDELC2 mRNA Expression Correlated with WHO Classification, IDH1 Status, and 1p/19q Co-Deletion of Gliomas in the CGGA Database

To determine the possible role of KDELC2 in glioma patients from different countries, we evaluated the association between KDELC2 mRNA expression and some clinicopathological parameters from the CGGA. In this study, we noticed that KDELC2 expression correlated with tumor grades (Supplementary Figure S1). Further, IDH1 wild-type and chromosome 1p/19q non-co-deletion gliomas had significantly higher KDELC2 mRNA expression than mutated IDH1 and chromosome 1p/19q tumors (Supplementary Figure S1). Therefore, KDELC2 also played an oncogenic role on gliomas in Chinese patients.

### 3.3. KDELC2 Overexpression in GBM Cell Lines

**Figure 2:**
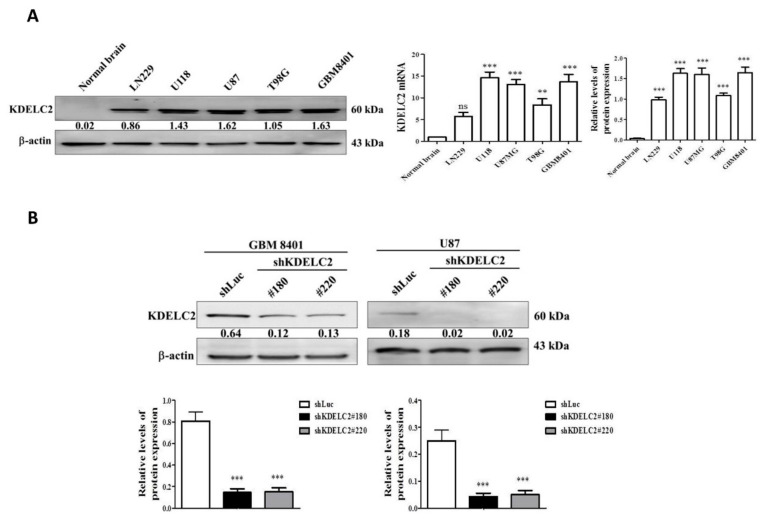
KDELC2 expression correlated with glioblastoma development. (**A**) High KDELC2 mRNA and protein expression in GBM cell lines. Bars, means ± SEM. ** *p* < 0.01; *** *p* < 0.0001; *p* = ns, non-significant. (**B**) The shKDELC2-transfected GBM8401 and U87 suppressed KDELC2 protein expression.

The qRT-PCR and WB assays were performed to assess for KDELC2 expression in GBM cell lines and normal brain tissue cDNA and lysates. Of all GBM cell lines, GBM8401, U87, U118, and T98G had higher KDELC2 mRNA and protein expression than the normal brain tissues (Figure 2A).

### 3.4. KDELC2 Knockdown Suppressed Tumor Proliferation in GBMs

**Figure 3:**
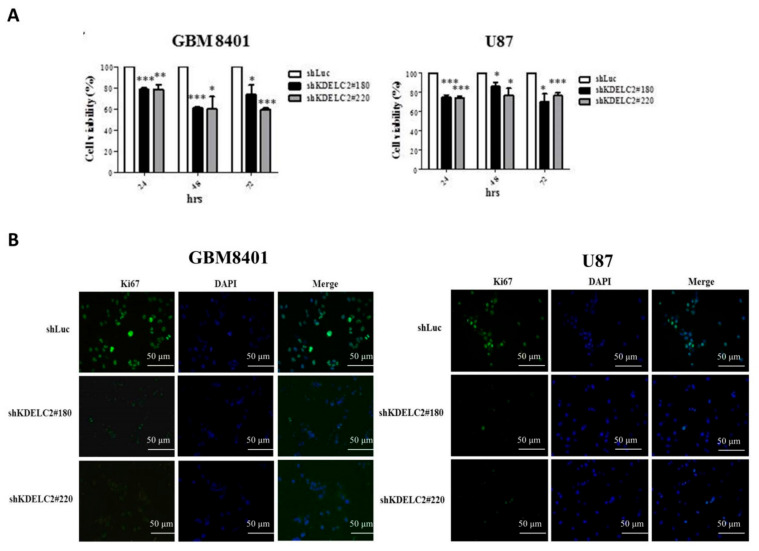
KDELC2 knockdown impacted GBM proliferation. (**A**) GBM with shKDELC2 transfection inhibited tumor viability. Bars, means ± SEM. * *p* < 0.05; ** *p* < 0.01; *** *p* < 0.0001. (**B**) Immunofluorescence assay confirmed that knockdown of KDELC2 decreased the proliferative index.

First, we evaluated the efficiency of KDELC2 knockdown by transfecting shKDELC2#180 and shKDELC2#220 on GBM8401 and U87 GBMs. The results of qRT-PCR and WB assays revealed significantly decreased KDELC2 expression in shKDELC2-transfected GBM cells (Figure 2B). Compared with GBM8401 and U87 GBMs transfecting shRNA with luciferase vector (shLuc), our data revealed significantly decreased tumor viability of the above GBMs after shKDELC2#180 and shKDELC2#220 transfection in 24, 48, and 72 h (Figure 3A). GBM8401 and U87 GBMs with shKDELC2 transfection had lower Ki67 IF staining than the shLuc group (Figure 3B). Therefore, we concluded that KDELC2 knockdown could inhibit cell viability by downregulating the proliferative activity of GBMs. 

### 3.5. KDELC2 Knockdown Interrupted Cell Cycle of GBMs

**Figure 4:**
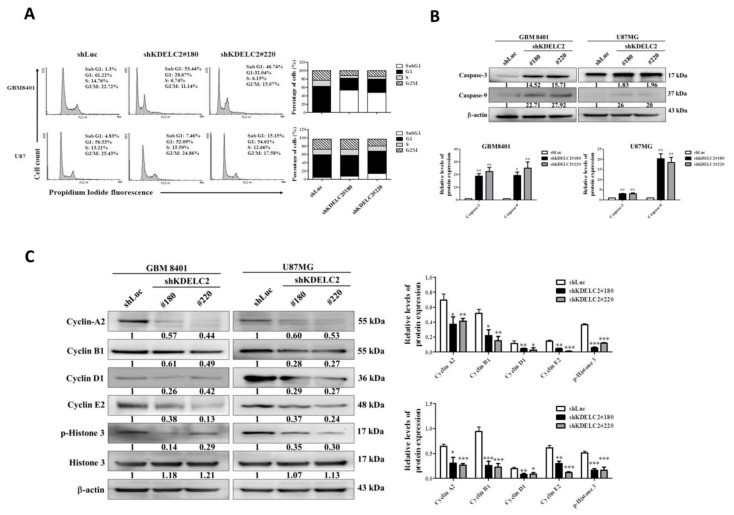
KDELC2 knockdown interrupted cell cycle in glioblastomas (GBMs). (**A**) Flow cytometry assay showed an increase in the sub-G1 phase of GBMs after shKDELC2 transfection. (**B**) Knockdown of KDELC2 increased caspase 3 and caspase 9 expression. (**C**) Inhibition of cell cycle checkpoint expression in shKDELC2-transfected GBMs. Bars, means ± SEM. * *p* < 0.05; ** *p* < 0.01; *** *p* < 0.0001.

As the results of flow cytometry showed a significantly higher percentage of the sub-G1 phase of GBM8401 and U87 GBMs with shKDELC2 transfection (Figure 4A), KDELC2 knockdown effectively induced GBM cell cycle delay. WB analysis showed enhancement of caspase 3 and 9 expression after KDELC2 knockdown (Figure 4B). We also evaluated the relationship of KDELC2 and cell cycle checkpoint expression. Our data showed that KDELC2 knockdown downregulated cyclin-A2, cyclin-D1, cyclin-E2, and phosphorylated histone-3 (Figure 4C). In contrast, KDELC2 overexpression promoted tumor proliferation by upregulating cell cycle checkpoints.

### 3.6. KDELC2 Knockdown Inhibited Tumor Migration and Invasion of GBMs by Downregulating Matrix Metalloproteinase-2 (MMP2) Expression 

**Figure 5:**
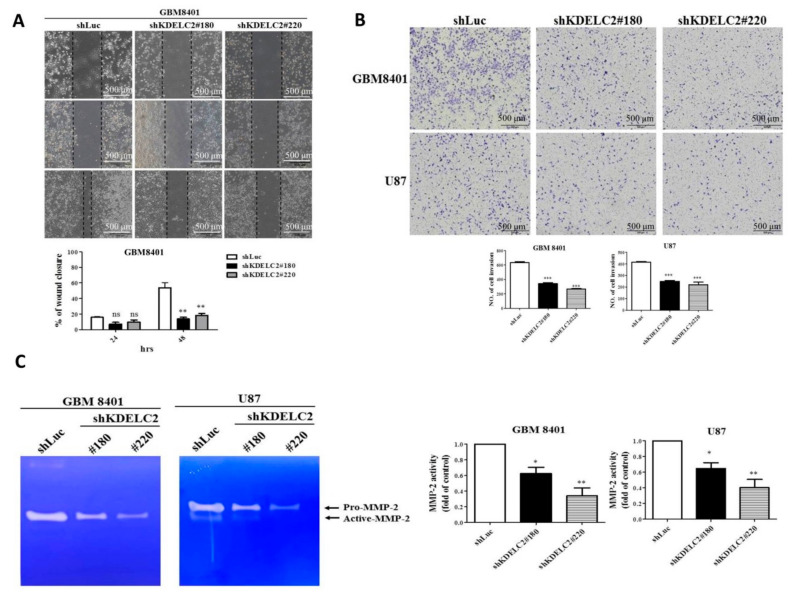
KDELC2 knockdown decreased the tumor-aggressive behavior of glioblastomas (GBMs). (**A**) Knockdown of KDELC2 suppressed GBM migration and (**B**) invasion ability after 48 h of culture. (**C**) Results of gelatin zymography revealed the inhibition of MMP2 after shKDELC2 transfection. Bars, means ± SEM. * *p* < 0.05; ** *p* < 0.01; *** *p* < 0.0001; ns, non-significant.

A wound healing test and transmembrane invasion assay were performed to evaluate tumor behaviors. The results showed that both GBM8401 and U87 with KDELC2 knockdown had lower ability of tumor migration within 48 h (Figure 5A; Supplementary Figure S2). In addition, compared with shLuc-transfected GBMs, significantly less tumor cells could penetrate through the transmembrane barrier in the aforementioned GBMs with shKDELC2 transfection (Figure 5B). As the cell–matrix interaction plays an important role on tumor migration and invasion, gelatin zymography and WB analysis were performed to explore the possible factors that could induce KDELC2 expression. The knockdown of KDELC2 inhibited the expression of MMP-2 in GBM8401 and U87 GBMs (Figure 5C; Supplementary Figure S3). Therefore, KDELC2 expression could activate tumor migration and invasion by upregulating MMP2 expression in GBMs.

### 3.7. KDELC2 Knockdown Suppressed 3D Tumor Spheroid Formation and Stemness Factors’ Expression of GBMs

**Figure 6:**
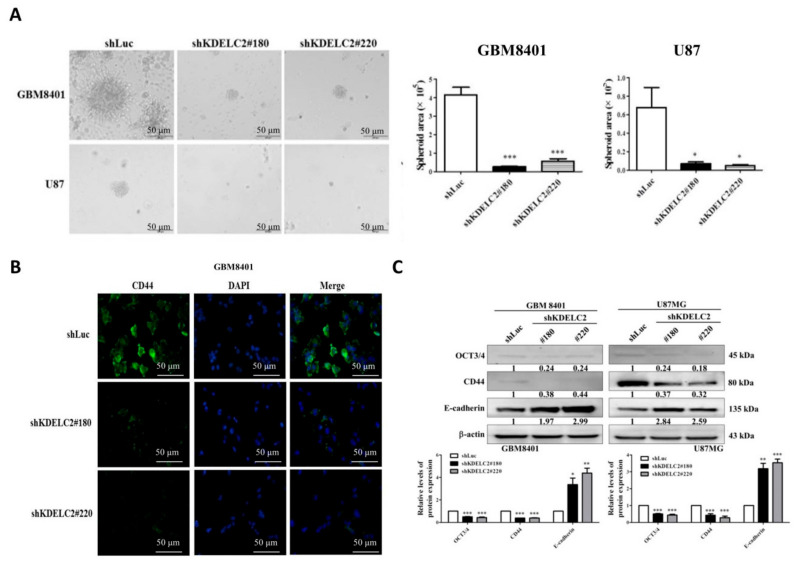
KDELC2 knockdown increased tumor stemness of glioblastomas (GBMs). (**A**) Knockdown of KDELC2 influenced the ability of tumor sphere formation in GBMs. (**B**) Downregulation of CD44 was noted on the GBM8401 cell line with shKDELC2 transfection. (**C**) Western blotting revealed that KDELC2 knockdown decreased OCT3/4 and CD44 and increased the E-cadherin protein expression in GBM8401 and U87 GBM cells. Bars, means ± SEM. * *p* < 0.05; ** *p* < 0.01; *** *p* < 0.0001.

Glioma stem cells (GSCs) have radiotherapy and chemotherapy resistance and a high recurrence rate [19]. The characteristics of GSCs included tumor sphere formation and stemness [20,21]. Our results showed that KDELC2 knockdown of GBM8401 and U87 had smaller spheroid areas than the shLuc group of GBMs after 11 and 17 days of cell culture, respectively (Figure 6A). The IF analysis of CD44 showed higher expression in GBM without KDELC2 knockdown (Figure 6B; Supplementary Figure S4). Similarly, lower CD44 and OCT3/4 protein expression was also identified in GBM8401 and U87 GBM cells with shKDELC2 transfection than in those with shLuc transfection (Figure 6C). Furthermore, qRT-PCR showed that GBMs with KDELC2 knockdown significantly downregulated the mRNA expression of some stemness factors, including CD133, SOX-2, Nanog, and pou5f1 (Supplementary Figure S5). Therefore, KDELC2 effectively induced tumor stemness factors of GBMs. 

### 3.8. KDELC2 Knockdown Inhibited Epithelial–Mesenchymal Transition of GBMs

**Figure 7:**
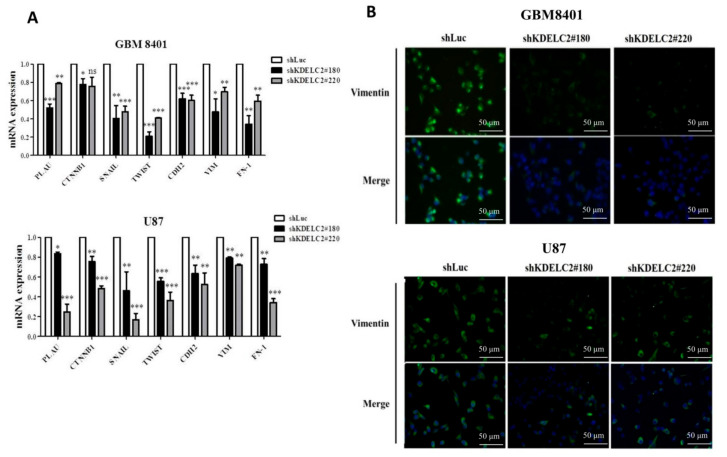
KDELC2 knockdown decreased epithelial–mesenchymal transition (EMT) of glioblastomas (GBMs). (**A**) Assay of qRT-PCR of GBM8401 revealed that knockdown of KDELC2 inhibited the mRNA expression of some mesenchymal factors, including PLAU, CTNNB1, Snail, Twist, CDH2, vimentin, and FN-1. Bars, means ± SEM. * *p* < 0.05; ** *p* < 0.01; *** *p* < 0.0001. (**B**) Immunofluorescence staining indicated that knockdown of KDELC2 decreased vimentin expression of GBM8401 and U87 GBMs.

As the epithelial–mesenchymal transition (EMT) is an important character of metastasis, we evaluated the EMT-related factors on GBMs with or without KDELC2 knockdown by performing qRT-PCR examination. Our results showed lower PLAU, CTNNB1, Snail, Twist, CDH2, vimentin, and FN-1 expression in shKDELC2-transfected GBM8401 and U87 GBMs than in shLuc-transfected GBMs (Figure 7A). From the viewpoint of IF expression, compared with shLuc-transfected GBM8401 and U87, lower vimentin was detected on GBMs with KDELC2 knockdown (Figure 7B). 

### 3.9. Downregulation of KDELC2 Suppressed GBM Angiogenesis

**Figure 8:**
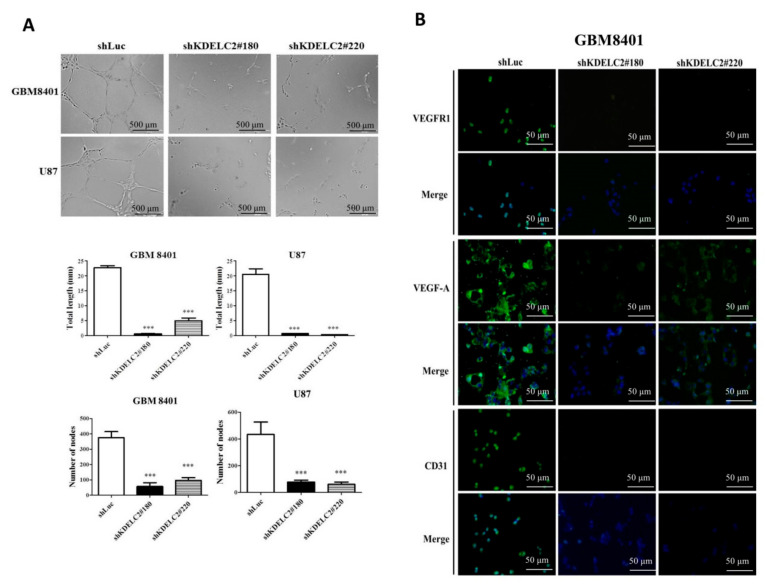
KDELC2 knockdown inhibited glioblastoma (GBM) angiogenesis. (**A**) HUVECs with supernatants from cultured GBM8401 with shKDELC2 transfection suppressed tube lengths and nodes. (**B**) Immunofluorescence staining images show decreased expression of VEGFR1, VEGFA, and CD31 in shKDELC2-transfected GBM8401. Bars, means ± SEM. * *p* < 0.05; ** *p* < 0.01; *** *p* < 0.0001.

To evaluate the impact of angiogenesis under KDELC2 knockdown, a HUVEC endothelial tube formation assay was performed on shLuc- and shKDELC2-transfected GBMs. First, KDELC2 knockdown of GBM8401 and U87 had significantly shorter endothelial cell tube lengths and lower node numbers than KDELC2 intact tumors (Figure 8A). The results of the IF study revealed that shKDELC2-transfected GBM8401 and U87 obviously downregulated the expression of VEGFR1, VEGFA, and CD31 (Figure 4A; Supplementary Figure S6). In summary, KDELC2 expression could promote the tumor angiogenesis of GBMs.

### 3.10. KDELC2 Knockdown Downregulated PI3k/mTOR/Akt, MAPK/ERK, and NF/kB Signaling Pathways by Suppression of Notch Receptor Expression

**Figure 9:**
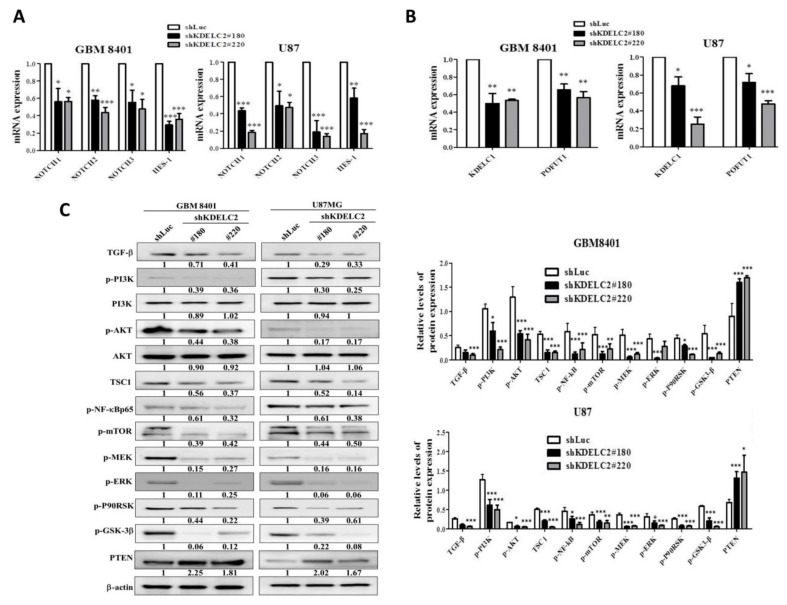
KDELC2 knockdown downregulated Notch signaling expression and PI3k/mTOR/Akt, MAPK/ERK, and NF/kB signaling pathways. (**A**) Knockdown of KDELC2 decreased Notch receptors 1–3, HES-1, and (**B**) KDELC1 and pofut 1 mRNA expression. (**C**) Western blot analysis proved that knockdown of KDELC2 inhibited TGF-β, NF-kB, and PI3K/mTOR/Akt signaling pathways. Bars, means ± SEM. * *p* < 0.05; ** *p* < 0.01; *** *p* < 0.0001.

KDELC2 knockdown not only inhibited Notch 1–3 mRNA expression but also decreased KDELC1 and pofut1 expression (Figure 9A,B). As the activated Notch receptors possibly upregulated the phosphoinositide 3-kinase (PI3k)/mTOR/Akt, mitogen-activated protein kinase (MAPK)/ERK, and nuclear factor kappa-light-chain-enhancer of activated B-cell (NF/kB) signaling pathways, we surveyed some representative factors of the above signaling pathways in shLuc and shKDELC2-transfected GBMs. Compared with the shLuc group of GBM8401 and U87 GBMs, our results demonstrated lower expression of phosphorylated PI3k (p-PI3k), phosphorylated mTOR (p-mTOR), phosphorylated Akt (p-Akt), phosphorylated MEK (p-MEK), phosphorylated ERK (p-ERK), and phosphorylated NF/kB p65 (p-NF/kB p65) and higher PTEN protein expression in the shKDELC2 GBMs (Figure 9C). Additionally, KDELC2 knockdown of GBM cell lines also showed suppression of transforming growth factor-β (TGF-β), p-GSK-3β, and p-90RSK expression (Figure 9C). In conclusion, KDELC2 overexpression could induce GBM growth by upregulating Notch, TGF-β, PI3k/mTOR/Akt, MAPK/ERK, and NF/kB signaling pathways.

### 3.11. KDELC2 Knockdown Promoted TMZ Cytotoxic Effect by Decreasing MGMT Expression

**Figure 10:**
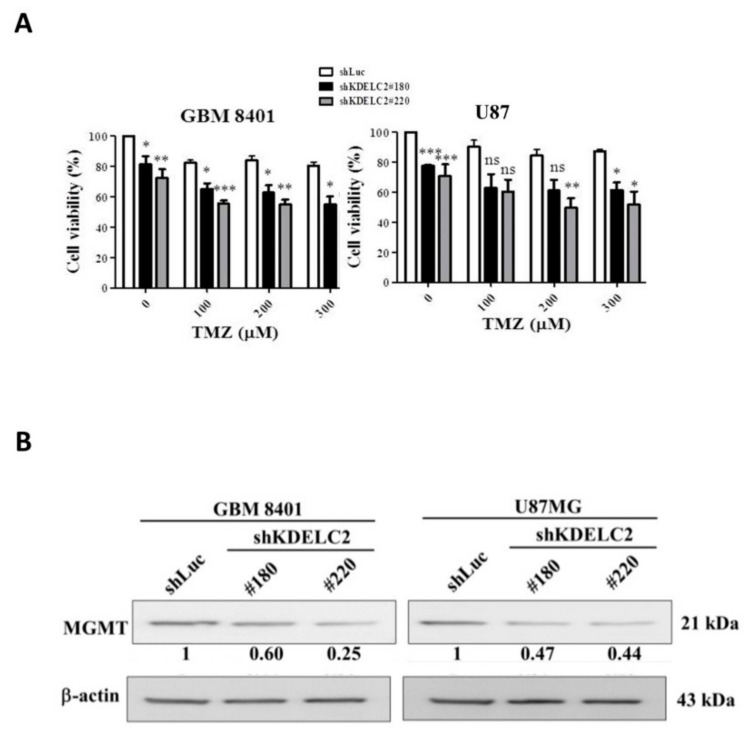
KDELC2 knockdown increased TMZ sensitivity by inhibiting MGMT expression. (**A**) Combination of shKDELC2 transfection and 300 µg TMZ had the least glioblastoma (GBM) viability. (**B**) Low MGMT expression was noted in shKDELC2-transfected GBM. Bars, means ± SEM. * *p* < 0.05; ** *p* < 0.01; *** *p* < 0.0001.

To determine the role of KDELC2 for TMZ therapeutic efficacy, we performed an MTT assay between shLuc and shKDELC2-transfected GBM8401 and U87 cells after TMZ administration. In this study, both GBM8401 and U87 GBMs with shKDELC2 knockdown had lower tumor viability than shLuc-transfected GBMs under 100–300 ug/mL of TMZ treatment (Figure 10A). As MGMT plays an important role in the discontinuation of TMZ treatment of GBMs, KDELC2 knockdown effectively inhibited MGMT expression in GBM8401 and U87 GBMs (Figure 10B). 

### 3.12. KDELC2 Knockdown Inhibited Tumor Proliferation and Angiogenesis in Orthotropic Human GBM Xenograft Mouse Models

**Figure 11:**
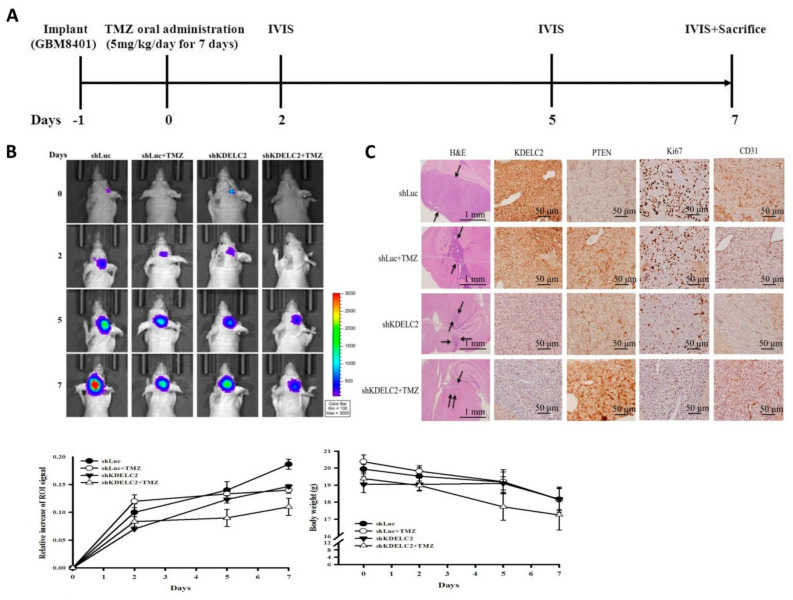
Effects of KDELC2 expression in orthotopic human GBM orthotropic xenograft mouse models. (**A**) The schedule of tumor implantation, in vivo imaging system (IVIS) measurement, and sacrifice. (**B**) IVIS assay showed that the shKDELC2 + TMZ group of mice had the least proliferative activity. (**C**) H&E and IHC stains of mice brain tumors revealed a smaller tumor area and lower Ki67, VEGFA, and CD31 expression levels in the shKDELC2 + TMZ group of mice (arrow indicates tumor-distributed areas).

To evaluate KDELC2 knockdown of GBMs in an in vivo study, we established orthotropic human GBM xenograft mouse models. Of all included mice, the group of shKDELC2 + TMZ showed the highest increase in ROI in the IVIS assay after 7 days of TMZ oral administration (Figure 11A,B). Our results implied that KDELC2 knockdown might aid the therapeutic benefit of TMZ, to some extent, in GBM mice. Based on the hematoxylin and eosin and IHC staining results of GBM tissues, smaller area of tumor growth, stronger PTEN staining, lower Ki67 proliferative activity, and lower CD31 expression were identified in the shKDELC2 + TMZ group than in the other three groups of mice, which indicated that the combination of KDELC2 knockdown and TMZ chemotherapy effectively inhibited tumor proliferation and angiogenesis of GBM cells (Figure 11C). According to the above-mentioned in vivo animal studies, KDELC2-targeted inhibitory drug and TMZ administration maybe a better therapeutic regimen for GBM than TMZ alone.

### 3.13. Higher KDELC2 Expression Correlated with Advanced Tumor Grades and Poor Prognosis in Human Glioma Tissue Microarrays

**Figure 12:**
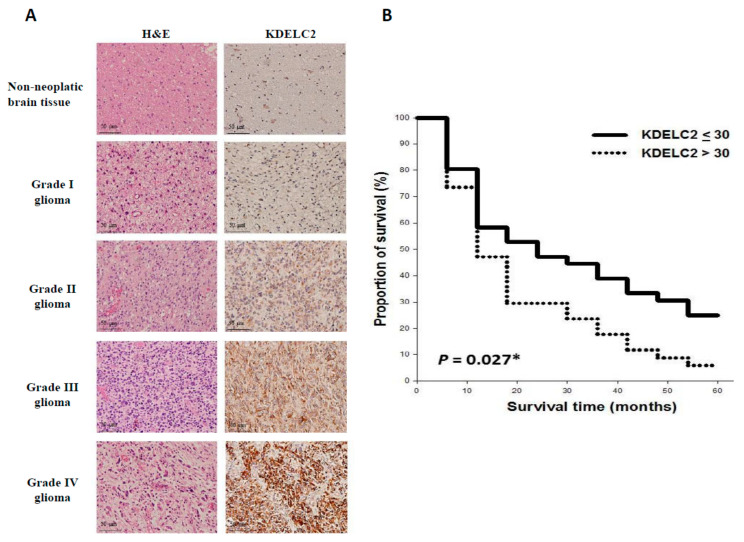
The association between KDELC2 expression with clinicopathologic parameters in human glioma tissues. (**A**) Higher KDELC2 IHC expression correlated with more advanced tumor grades of human glioma tissues. (**B**) Kaplan–Meier survival curve showing that high KDELC2 expression of human gliomas had significantly poor prognosis * *p* < 0.05.

To detect KDELC2 expression in human glioma tissues, we performed IHC to evaluate KDELC2 scores in tissue microarrays. Compared with non-neoplastic brain tissues, higher KDELC2 expression levels were identified in grade 2 to 4 gliomas (Figure 12A). KDELC2 IHC scores showed a positive correlation with tumor grades (Table 1). Of all included cases in this tissue microarray, grade 2–4 astrocytomas with IDH wild-type had relatively higher KDELC2 expression than the IDH mutant of astrocytic tumors (Table 1). Finally, we divided all human glioma cases into two groups: including high KDELC2 expression (KDELC2 IHC score > 30) and low KDELC2 expression (KDELC2 IHC score ≤ 30). The Kaplan–Meier analysis revealed that glioma patients with high KDELC2 expression had shorter overall survival time than those with low KDELC2 expression (Figure 12B). In this study, we successfully proved that KDELC2 overexpression might be associated with tumor development and poor prognosis in gliomas. We also performed a multivariate analysis to detect the related risk factors of the overall survival time in glioma patients. Patients’ age, KDELC2 expression, IDH1, ATRX, neurofilament, p-AxL, Nur77, H3Lys27, and PDGFRA were associated with glioma patients’ overall prognosis (Table 2).

## 4. Discussion

The mammalian Notch signaling pathway can be regulated by *O*-glucosylation of EGF repeats of Notch receptors [16]. Takeuchi et al. [11] demonstrated the transfer of *O*-glucose to Notch 1 receptor EGF 11 and Notch 3 EGF10 by either KDELC1 or KDELC2 but not to the Notch 2 receptor. In this study, we successfully proved that KDELC2 knockdown could interrupt KDELC1 expression, which implied that KDELC2 might have some regulations to KDELC1. Furthermore, KDELC2 knockdown could downregulate the Notch signaling pathway by inhibiting pofut 1 and Notch 1–3 receptor expression in GBMs, not just interfering with the post-translational modification of EGF repeats of NECD. Therefore, KDELC2 might play a critical role in the activation of the Notch signaling pathway in GBMs through the enhancement of Notch receptors.

The deregulation of the Notch signaling pathway could induce GSC maintenance and cancer cell characteristics, such as tumor proliferation, invasion, and migration [22]. Similarly, our results confirmed that KDELC2 induced GSC behaviors, such as tumor stemness, spheroid formation, and angiogenesis, depending on the activated Notch signaling pathway. We also confirmed that KDELC2 could induce NF/kB enrichment by the Notch signaling pathway. In conclusion, we discovered that KDELC2 was a critical factor related to several signaling pathways of gliomagenesis.

Of all GBMs, mesenchymal GSCs have the highest proliferative ability and poorest prognosis [23]. In the recent literature, mutated PTEN and NF1 can be viewed as molecular signatures of mesenchymal GBMs [24]. From in silico data, KDELC2 overexpression had a relatively high tendency of mesenchymal GBMs. Both loss of PTEN and NF1 enhancement were related to KDELC2 expression in our in vitro and human tissue assays. Moreover, our results proved that KDELC2 expression caused an increase in vimentin expression in GBMs. KDELC2 overexpression also promoted tumor metastasis by inducing EMT by upregulating TGF-β in GBMs.

TMZ resistance is attributed to therapeutic failure and poor prognosis in GBMs [25]. In the recent literature, MGMT methylation has been identified as the main factor for TMZ treatment failure [26]. In our study, knockdown of KDELC2 downregulated TMZ resistance by inhibiting MGMT protein expression. Therefore, we concluded that KDELC2 expression effectively developed the resistance to TMZ through MGMT dysregulation.

**Figure 13:**
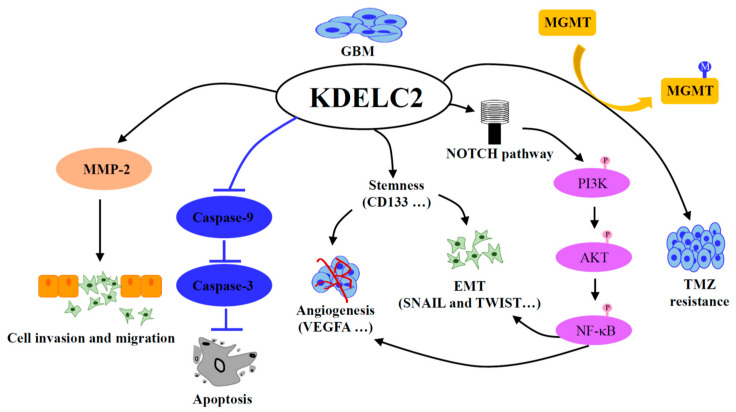
The illustration shows the impact of KDELC2 on cancer phenotype and molecular mechanism.

In conclusion, KDELC2 could induce GBM migration and invasion by stimulating MMP-2, and increased tumor proliferation through inhibiting Caspase 3 and 9 and accelerating cell cycle speed. In addition, overexpression of KDELC2 activated the NOTCH signaling cascade, including PI3K/Akt/mTOR and NF-kB pathways. Therefore, KDELC2 could induce GBM tumor stemness behaviors, such as angiogenesis and epithelial–mesenchymal transition by the activation of NF-kB. Finally, KDELC2 induced MGMT activation to result in TMZ resistance (Figure 13).

## Figures and Tables

**Table 1:** The correlation of KDELC2 IHC scores and World Health Organization (WHO) grades of gliomas.

	Number of Cases	Average Intensity	Average Tumor (%)	Average Score	Difference or Correlation *
Normal brain tissue	5	0.67	6.67	6.67	
Classification of gliomas					
Pilocytic astrocytoma	1	0	0	0	
Diffuse astrocytoma, IDH-mutant	2	0	0	0	Significant difference (*p* < 0.001 *)
Diffuse astrocytoma, IDH-WT	12	1.17	24.58	43.33	
Anaplastic astrocytoma, IDH-mutant	3	0.67	3.33	3.33	Significant difference (*p* = 0.012 *)
Anaplastic astrocytoma, IDH-WT	6	1.14	23.57	50.71	
Glioblastoma, IDH-mutant	4	1.33	17.5	31.67	Significant difference (*p* = 0.039 *)
Glioblastoma, IDH-WT	33	1.69	42.14	85.14	
Diffuse midline glioma, H3 K27M-mutant	8	1.5	42.86	72.86	
Oligodendroglioma, IDH-mutant	1	0	0	0	Not significant difference (*p* = 1)
Oligodendroglioma, IDH-WT	2	0	0	0	
Anaplastic oligodendroglioma, IDH-mutant	2	0.25	13.75	13.75	No significant difference (*p* = 0.258)
Anaplastic oligodendroglioma, IDH-WT	2	1.5	32.5	57.5	
**WHO grades of gliomas**					
WHO grade I	1	0	0	0	Positive correlation (*p* < 0.001 ^#^)
WHO grade II	17	0.83	16.67	29.17	Positive correlation (*p* < 0.001 ^#^)
WHO grade III	13	0.88	18.44	33.44	
WHO grade IV	45	1.6	39.64	76.18	

* The difference was analyzed by a Paired *t*-test; ^#^ The correlation was analyzed by the Pearson Product Method Correlation test.

**Table 2:** Univariate and multivariate analysis of risk factors associated with KDELC2 expression.

Variable	Total	Univariate Analysis		Multivariate Analysis	
		OR (95% CI)	*p*-Value	OR (95% CI)	*p*-Value
Sex					
Male	47	1			
Female	29	1.56 (1.48–1.73)	0.064	1.61 (1.51–1.84)	0.116
Age					
<50	36	1			
≥50	40	2.64 (1.70–8.90)	0.017 *	1.99 (1.54–3.83)	0.100
IDH1 R132H					
Negative	63	1			
Positive	13	0.25 (0.11–0.62)	0.009 *	0.27 (0.10–0.65)	0.109
ATRX					
Preserve	39	1			
Loss of expression	37	0.83 (0.69–0.81)	0.276	0.70 (0.59–0.76)	0.116
H3K27M					
Negative	67	1			
Positive	9	1.26 (0.79–1.53)	0.485	1.23 (0.77–1.49)	0.212
MGMT					
Preserved	43	1			
Loss of expression	33	1.10 (1.05–1.22)	0.678	1.08 (1.04–1.15)	<0.001 *
EGFR					
Negative	69	1			
Positive	7	1.55 (0.94–1.90)	0.228	1.52 (1.10–1.86)	0.357
EGFRvIII					
Negative	58	1			
Positive	18	2.28 (2.17–2.52)	<0.001 *	2.49 (2.29–2.92)	0.004 *
P53					
Negative	36	1			
Overexpression	40	1.19 (1.10–1.35)	0.482	1.11 (1.05–1.25)	0.034 *
Neurofilament					
Negative	59	1			
Positive	17	0.59 (0.18–0.85)	0.126	0.62 (0.18–0.87)	0.253
NF1					
Negative	42	1			
Positive	34	1.58 (1.42–1.89)	0.063	1.51 (1.37–1.85)	0.001 *
AxL					
Negative	29	1			
Positive	47	2.11 (1.58–4.58)	0.008 *	1.95 (1.46–4.46)	<0.001 *
p-AxL					
Negative	22	1			
Positive	54	1.36 (1.01–3.09)	0.354	1.29 (0.98–2.90)	0.013 *
NUR77					
Negative	27	1			
Positive	49	1.89 (1.45–3.70)	0.024 *	2.13 (1.59–4.99)	0.006 *
H3Lys27					
Preserved	67	1			
Loss of expression	9	3.25 (1.33–9.98)	0.039 *	6.08 (1.54–14.36)	0.001 *
PDGFRA					
Negative	5	1			
Positive	71	0.71 (0.23–1.27)	0.528	1.43 (1.25–5.25)	0.721

* means statistical significance.

## Supplementary Materials

The following are available online at https://www.mdpi.com/2227-9059/8/9/339/s1. Figure S1: The association of KDELC2 expression with tumor grades and genetic mutation of gliomas in Chinese Glioma Genome Atlas database. Figure S2: The impact of glioma migration after KDELC2 knockdown. Figure S3: The influence of MMP2 expression by the suppression of KDELC2. Figure S4: IF staining of CD44 in U87 cells after KDELC2 inhibition. Figure S5: Knockdown of KDELC2 interrupted the stemness factors expression. Figure S6: The suppression of angiogenetic factors by KDELC2 knockdown in U87 cells.
