# Supplementary material for: Molecular Mechanisms of KDELC2 on Glioblastoma Tumorigenesis and Temozolomide Resistance
Source: Biomedicines. 2020 Sep 10;8(9):339. doi: 10.3390/biomedicines8090339 (PMC7555920; doi:10.3390/biomedicines8090339)
Supplement: Supplementary file 1 [file biomedicines-08-00339-s001.zip › biomedicines-910361-supplementary final/Biomedicines-910361-Supplementary Figure legends.docx]

Supplementary Figure legends

Figure S1. From the Chinese Glioma Genome Atlas database, KDELC2 mRNA expression positively correlated with tumor grades, IDH-WT, and 1p/19q co-deletion of gliomas. Bars, means ± SEM. * *P* < 0.05; ** *P* < 0.01; *** *P* < 0.0005; **** *P* < 0.0001; ns, non-significant.

Figure S2. The wound healing assay revealed that shKDELC2-transfected U87 cells had lower migration ability after 48 h. Bars, means ± SEM. ** *P* < 0.01; ns, non-significant.

Figure S3. Both GBM8401 and U87 GBM with shKDELC2 transfection significantly decreased MMP2 expression. Bars, means ± SEM. * *P* < 0.05; ** *P* < 0.01; *** *P* < 0.0005; **** *P* < 0.0001; ns, non-significant.

Figure S4. IF staining showed that the knockdown of KDELC2 in U87 cells inhibited CD44 expression.

Figure S5. GBM8401 and U87 with shKDELC2 transfection had lower stemness-related mRNA expression than shLuc transfection. Bars, means ± SEM. * *P* < 0.05; ** *P* < 0.01; *** *P* < 0.0005; **** *P* < 0.0001; ns, non-significant.

Figure S6. In U87 cells, shKDELC2 group showed lower VEGFR1, VEGFA, and CD31 IF stain than shLuc-transfected tumors.
