# Supplementary material for: Molecular Mechanisms of KDELC2 on Glioblastoma Tumorigenesis and Temozolomide Resistance
Source: Biomedicines. 2020 Sep 10;8(9):339. doi: 10.3390/biomedicines8090339 (PMC7555920; doi:10.3390/biomedicines8090339)
Supplement: Supplementary file 1 [file biomedicines-08-00339-s001.zip › biomedicines-910361-supplementary final/Table S1.docx]

Table S1. The information of qRT/PCR primer sequences of related genes

| Gene name |  | Sequence (5'->3') |
| --- | --- | --- |
| *CDH1* | Forward primer | AGGCCAAGCAGCAGTACAT |
|  | Reverse primer | AAATGTGTCTGGCTCCTGGG |
| *Snail* | Forward primer | GGTTCTTCTGCGCTACTGCT |
|  | Reverse primer | TAGGGCTGCTGGAAGGTAAA |
| *ZEB-2* | Forward primer | AAGCCAGGGACAGATCAGC |
|  | Reverse primer | GCCACACTCTGTGCATTTG |
| *CTNNB1* | Forward primer | GAAACGGCTTTCAGTTGAGC |
|  | Reverse primer | CTGGCCATATCCACCAGAGT |
| *CD133* | Forward primer | CAGAGTACAACGCCAAACCA |
|  | Reverse primer | AAATCACGATGAGGGTCAGC |
| *FN-1* | Forward primer | CCAACCTACGGATGACTCGT |
|  | Reverse primer | GCTCATCATCTGGCCATTTT |
| *NES* | Forward primer | AACAGCGACGGAGGTCTCTA |
|  | Reverse primer | TTCTCTTGTCCCGCAGACTT |
| *TWIST1* | Forward primer | GTCCGCAGTCTTACGAGGAG |
|  | Reverse primer | CCAGCTTGAGGGTCTGAATC |
| *CDH2* | Forward primer | GACAATGCCCCTCAAGTGTT |
|  | Reverse primer | CCATTAAGCCGAGTGATGGT |
| *VIM* | Forward primer | TGCCCTTAAAGGAACCAATG |
|  | Reverse primer | TCCAGCAGCTTCCTGTAGGT |
| *PLAU* | Forward primer | GCCATCCCGGACTATACAGA |
|  | Reverse primer | ACACAGCATTTTGGTGGTGA |
| *POU5F1* | Forward primer | GGGAGATTGATAACTGGTGTGTT |
|  | Reverse primer | GTGTATATCCCAGGGTGATCCTC |
| *NANOG* | Forward primer | GGACACTGGCTGAATCCTTCC |
|  | Reverse primer | CTCGCTGATTAGGCTCCAACC |
| *SOX-2* | Forward primer | TACAGCATGTCCTACTCGCAG |
|  | Reverse primer | GAGGAAGAGGTAACCACAGGG |
| *Grp78* | Forward primer | CACGTCCAACCCGGAGAA |
|  | Reverse primer | TTCCAAGTGCGTCCGATGA |
| *POFUT1* | Forward primer | CTGATGACCCGATGGTAAGC |
|  | Reverse primer | AAGCCTCCTTTCACCAACCT |
| *CHOP* | Forward primer | CTCTGACTGGAATCTGGAGAGTG |
|  | Reverse primer | CTGAGTCATTGCCTTTCTCCTTCG |
| *PERK* | Forward primer | ACGATGAGACAGAGTTGCGAC |
|  | Reverse primer | ATCCAAGGCAGCAATTCTCCC |
| *KDELC1* | Forward primer | ATTCAGGCAGTGGATACATCAG |
|  | Reverse primer | TGAAGGACCCATCTTTTCGG |
| *NOTCH1* | Forward primer | AAGCTGCATCCAGAGGCAAAC |
|  | Reverse primer | TGGCATACACACTCCGAGAACAC |
| *NOTCH3* | Forward primer | GAGCCAATGCCAACTGAAGAG |
|  | Reverse primer | GGCAGATCAGGTCGGAGATG |
| *NOTCH2* | Forward primer | ACAGTTGTGTCTGCTCACCAGGAT |
|  | Reverse primer | GCGGAAACCATTCACACCGTTGAT |
| *NOTCH4* | Forward primer | AGTCCAGGCCTTGCCAGAACG |
|  | Reverse primer | GTAGAAGGCATTGGCCAGAGAG |
| *HES-1* | Forward primer | GGACATTCTGGAAATGACAGTGA |
|  | Reverse primer | AGCACACTTGGGTCTGTGCTC |
| *sXBP1* | Forward primer | CTGAGTCCGAATCAGGTGCAG |
|  | Reverse primer | ATCCATGGGGAGATGTTCTGG |
| *ATF4* | Forward primer | GTTCTCCAGCGACAAGGCTA |
|  | Reverse primer | ATCCTGCTTGCTGTTGTTGG |
| *Grp78/BiP* | Forward primer | TGTTCAACCAATTATCAGCAAACTC |
|  | Reverse primer | TTCTGCTGTATCCTCTTCACCAGT |
| *EDEM1* | Forward primer | CAAGTGTGGGTACGCCACG |
|  | Reverse primer | AAAGAAGCTCTCCATCCGGTC |
| *LC3B* | Forward primer | GATGTCCGACTTATTCGAGAGC |
|  | Reverse primer | TTGAGCTGTAAGCGCCTTCTA |
| *ATG4b* | Forward primer | ATGGACGCAGCTACTCTGAC |
|  | Reverse primer | TTTTCTACCCAGTATCCAAACGG |
| *Aatg5* | Forward primer | AAAGATGTGCTTCGAGATGTGT |
|  | Reverse primer | CACTTTGTCAGTTACCAACGTCA |
| *SQSTM1* | Forward primer | GCACCCCAATGTGATCTGC |
|  | Reverse primer | CGCTACACAAGTCGTAGTCTGG |
| *ATG12* | Forward primer | AACAAAGAAATGGGCTGTGGAGCG |
|  | Reverse primer | TTCCGAGGCCACCAGTTTAAGGAA |
| *KDELC2* | Forward primer | TGGAGCATCGAAAAGTCAATGG |
|  | Reverse primer | CATACGTTGGAAGGACAACATCT |
| *GAPDH* | Forward primer | GCACCGTCAAGGCTGAGAAC |
|  | Reverse primer | ATGGTGGTGAAGACGCCAGT |
