# Supplementary material for: Molecular Mechanisms of KDELC2 on Glioblastoma Tumorigenesis and Temozolomide Resistance
Source: Biomedicines. 2020 Sep 10;8(9):339. doi: 10.3390/biomedicines8090339 (PMC7555920; doi:10.3390/biomedicines8090339)
Supplement: Supplementary file 1 [file biomedicines-08-00339-s001.zip › biomedicines-910361-supplementary final/Table S2.docx]

Table S2. The information of included antibodies for western-blot analysis

| **Antibody** | **Company** | **Host** | **Dilution** |
| --- | --- | --- | --- |
| KDELC2 | Thermo Fisher Scientific | Rabbit | 1:500 |
| β-Actin | Millpore | Mouse | 1:1000 |
| Cleaved Caspase-3 (D175) (5A1E) | Cell signaling | Rabbit | 1:500 |
| Cleaved Caspase-9 (D315) | Cell signaling | Rabbit | 1:500 |
| Cyclin A2 (BF683) | Cell signaling | Rabbit | 1:1000 |
| Cyclin B1 (D5C10) | Cell signaling | Rabbit | 1:1000 |
| Cyclin D1 (92G2) | Cell signaling | Rabbit | 1:1000 |
| Cyclin E2 | Cell signaling | Rabbit | 1:1000 |
| P-Histon H3 (S10) (D2C8) | Cell signaling | Rabbit | 1:1000 |
| Histon H3 | Cell signaling | Rabbit | 1:1000 |
| MMP-2 | Spring Bioscience | Rabbit | 1:500 |
| Oct3/Oct-4 | Spring Bioscience | Rabbit | 1:1000 |
| CD44 | Thermo Fisher Scientific | Mouse | 1:1000 |
| E-cadherin | Santa Cruz | Rabbit | 1:500 |
| TGF-β | Santa Cruz | Rabbit | 1:250 |
| P-PI3K-P85 (Y458)/P55 (Y199) | Cell signaling | Rabbit | 1:250 |
| PI3 Kinase P85 | Cell signaling | Rabbit | 1:500 |
| P-AKT (S473) | Cell signaling | Rabbit | 1:250 |
| AKT (C67E7) | Cell signaling | Rabbit | 1:500 |
| TSC1 | Genetex | Rabbit | 1:500 |
| NF-κBp65 | Santa Cruz | Rabbit | 1:1000 |
| p-mTOR | Abcam | Rabbit | 1:1000 |
| P-MEK1/2 (S217/221) (41G9) | Cell signaling | Rabbit | 1:1000 |
| P-P44/42 MAPK (T202/Y204) (D13.14.4E) | Cell signaling | Rabbit | 1:1000 |
| P-P90RSK (S380) (D3H11) | Cell signaling | Rabbit | 1:250 |
| P-GSK-3-Beta (S9) (D85E12) | Cell signaling | Rabbit | 1:1000 |
| PTEN (138G6) | Cell signaling | Rabbit | 1:1000 |
| MGMT | Millpore | Mouse | 1:250 |
| LC3B | Sigma-Aldrich | Rabbit | 1:1000 |
| NLRP3 (D4D8T) | Cell signaling | Rabbit | 1:500 |
| IL-1β (3A6) | Cell signaling | Mouse | 1:500 |
| Caspase-1 (D7F10) | Cell signaling | Rabbit | 1:500 |
