# Supplementary material for: Molecular Mechanisms of KDELC2 on Glioblastoma Tumorigenesis and Temozolomide Resistance
Source: Biomedicines. 2020 Sep 10;8(9):339. doi: 10.3390/biomedicines8090339 (PMC7555920; doi:10.3390/biomedicines8090339)
Supplement: Supplementary file 1 [file biomedicines-08-00339-s001.zip › biomedicines-910361-supplementary final/Table S3.docx]

Table S3. The information of included antibodies for IF assay

| **Antibody** | **Company** | **Country** | **Host** | **Dilution** |
| --- | --- | --- | --- | --- |
| Ki67 | GeneTex | USA | Rabbit | 1:100 |
| VEGFR1 | Abcam | UK | Rabbit | 1:100 |
| VEGFA | Abcam | UK | Mouse | 1:200 |
| CD31 | Abcam | UK | Rabbit | 1:20 |
| Vimentin | Dako | USA | Rabbit | 1:50 |
| E-cadherin | Dako | USA | Rabbit | 1:20 |
| CD44 | Thermo Fisher Scientific | USA | Mouse | 1:100 |
| LC3B | Sigma-Aldrich | USA | Rabbit | 1:1000 |
| HIF-1a | Abcam | UK | Rabbit | 1:20 |
