# Supplementary material for: Molecular Mechanisms of KDELC2 on Glioblastoma Tumorigenesis and Temozolomide Resistance
Source: Biomedicines. 2020 Sep 10;8(9):339. doi: 10.3390/biomedicines8090339 (PMC7555920; doi:10.3390/biomedicines8090339)
Supplement: Supplementary file 1 [file biomedicines-08-00339-s001.zip › biomedicines-910361-supplementary final/Table S4.docx]

Table S4. The information of included antibodies for IHC analysis.

| **Antibody** | **Company** | **Country** | **Host** | **Dilution** |
| --- | --- | --- | --- | --- |
| KDELC2 | Thermo Fisher Scientific | USA | Rabbit | 1:500 |
| IDH1 R132H | Dianova | Germany | Mouse | 1:100 |
| MGMT | Thermo Fisher Scientific | USA | Mouse | 1:100 |
| EGFR | Thermo Fisher Scientific | USA | Mouse | 1:100 |
| EGFRvIII | Absolute | UK | Mouse | 1:100 |
| p53 | DAKO | USA | Mouse | 1:100 |
| AxL | Sigma-Aldrich | USA | Rabbit | 1:50 |
| p-AxL | R&D system | USA | Mouse | 1:50 |
| NUR77 | Abcam | UK | Rabbit | 1:100 |
| H3 K27me3 | Millipore | USA | Rabbit | 1:1000 |
| ATRX | ATLAS | Sweden | Rabbit | 1:100 |
| H3 K27M | Millipore | USA | Rabbit | 1:100 |
| Neurofilament | DAKO | USA | Mouse | 1:100 |
| PDGFRA | Santa Cruz | USA | Mouse | 1:100 |
| NF1 | Abcam | UK | Rabbit | 1:100 |
| VEGFR1 | Abcam | UK | Rabbit | 1:100 |
| VEGFA | Abcam | UK | Mouse | 1:200 |
| CD31 | Abcam | UK | Rabbit | 1:20 |
