# Supplementary figures and images for: Molecular Mechanisms of KDELC2 on Glioblastoma Tumorigenesis and Temozolomide Resistance
Source: Biomedicines. 2020 Sep 10;8(9):339. doi: 10.3390/biomedicines8090339 (PMC7555920; doi:10.3390/biomedicines8090339)

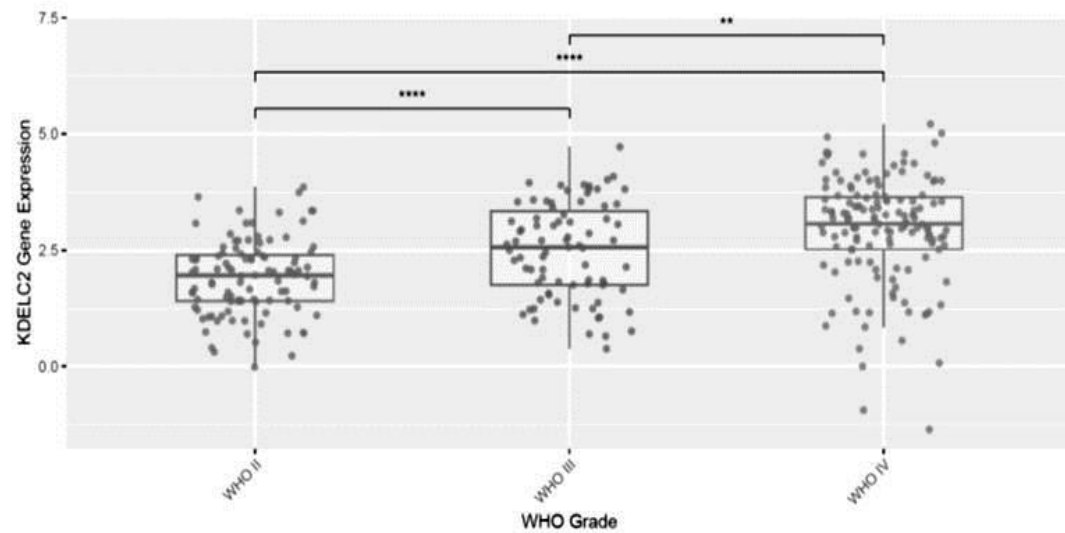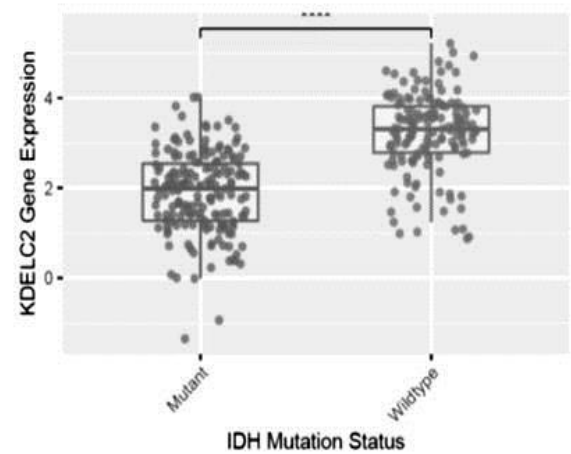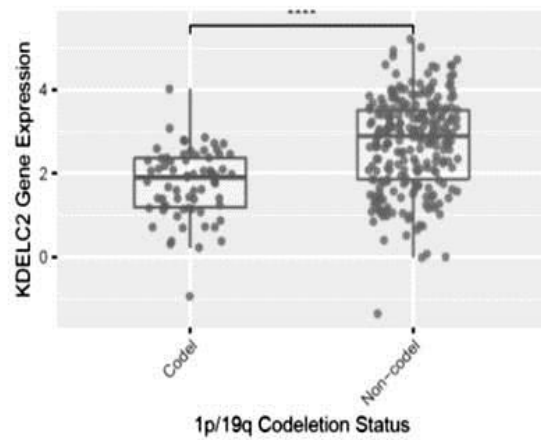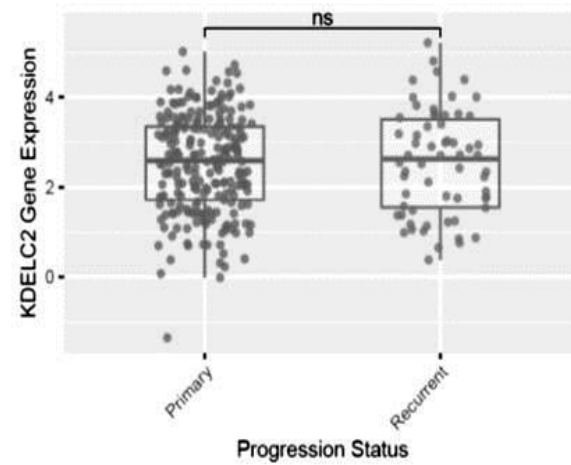

Supplement: Supplementary file 1 [file biomedicines-08-00339-s001.zip › biomedicines-910361-supplementary final/Fig. S1.pdf]

# U87

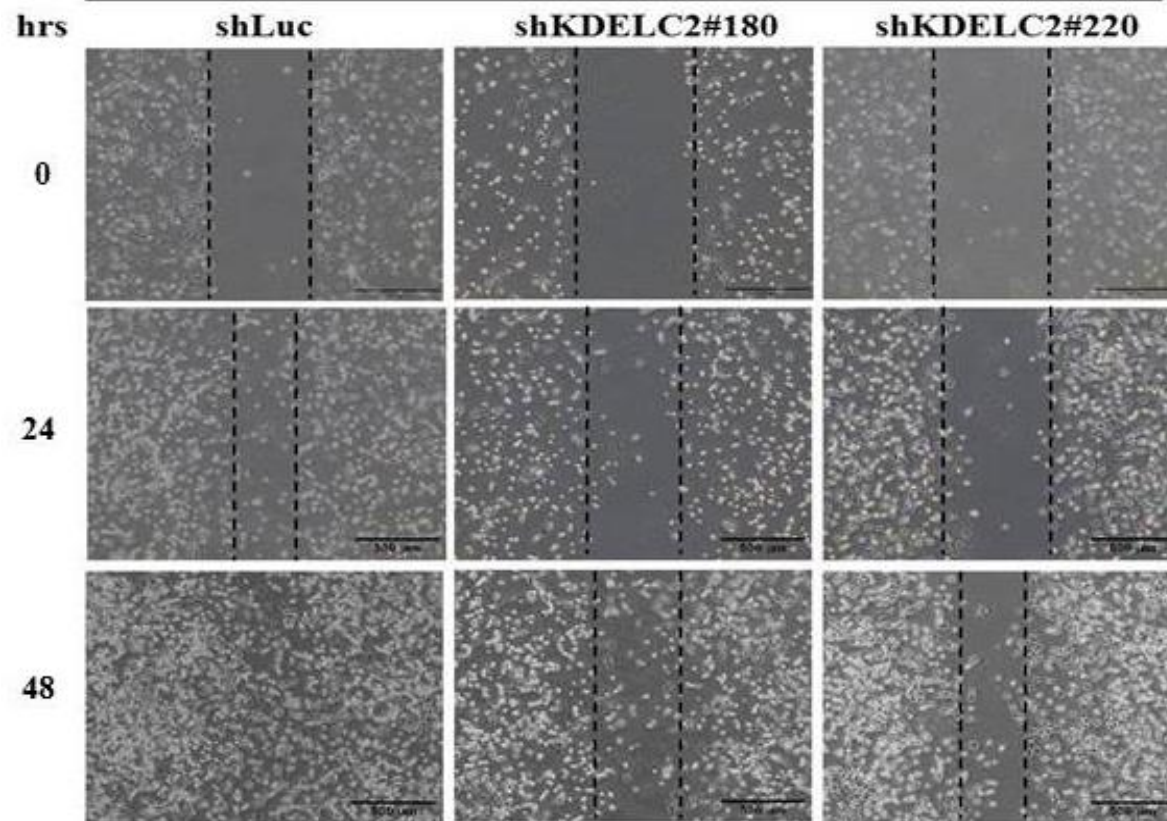

# U87

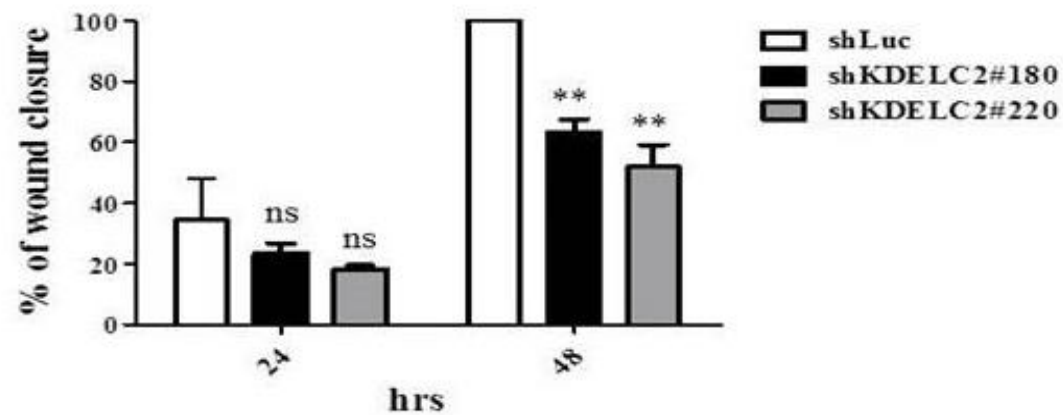

Supplement: Supplementary file 1 [file biomedicines-08-00339-s001.zip › biomedicines-910361-supplementary final/Fig. S2.pdf]

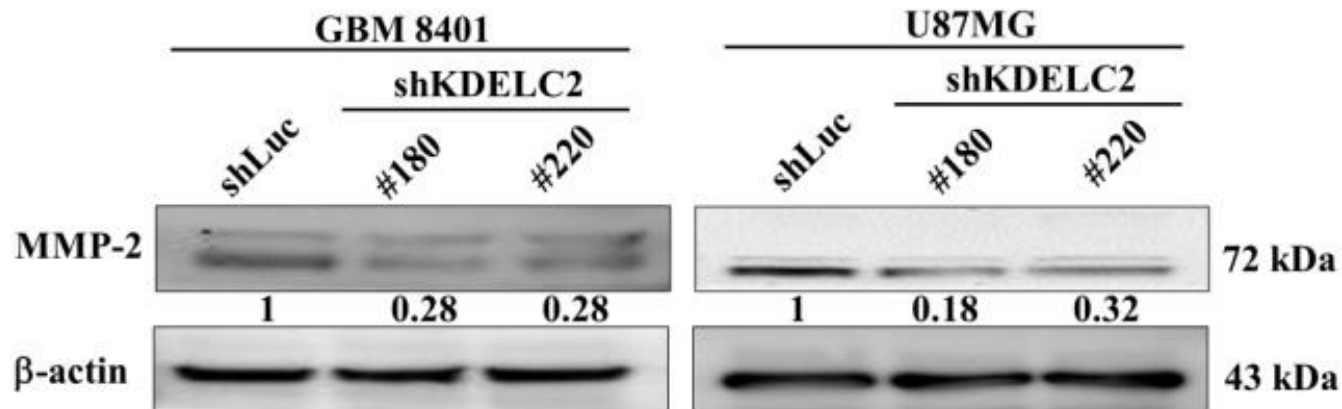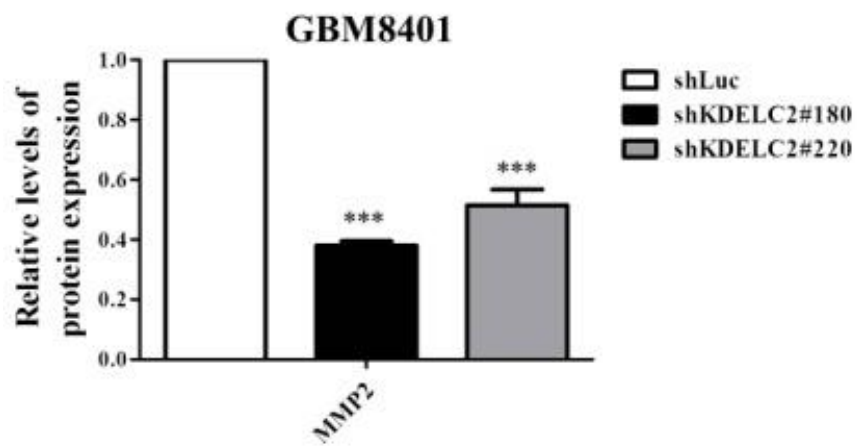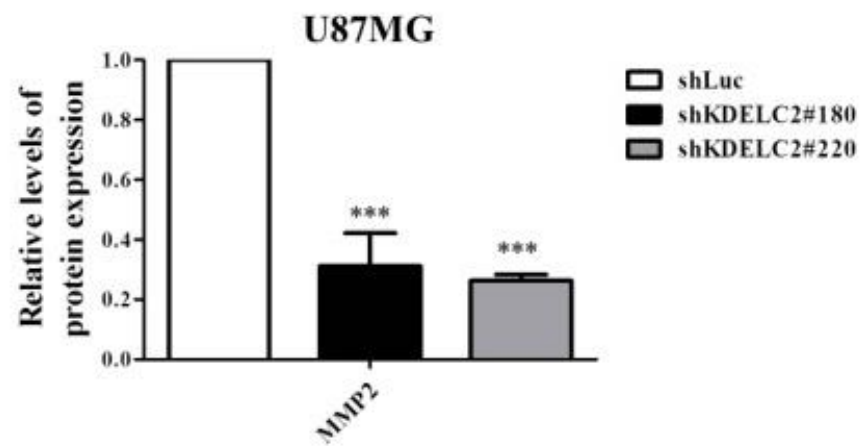

Supplement: Supplementary file 1 [file biomedicines-08-00339-s001.zip › biomedicines-910361-supplementary final/Fig. S3.pdf]

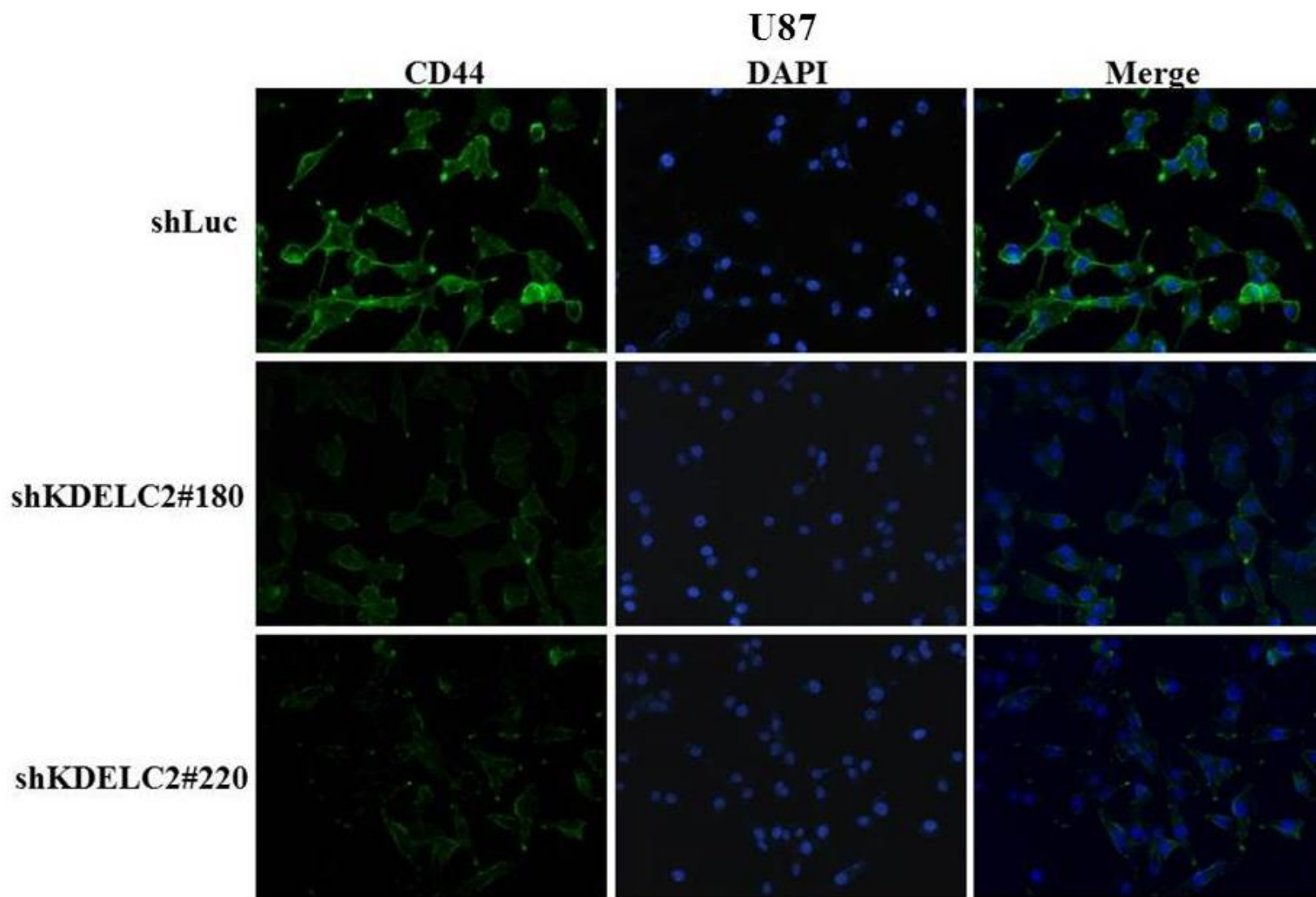

Supplement: Supplementary file 1 [file biomedicines-08-00339-s001.zip › biomedicines-910361-supplementary final/Fig. S4.pdf]

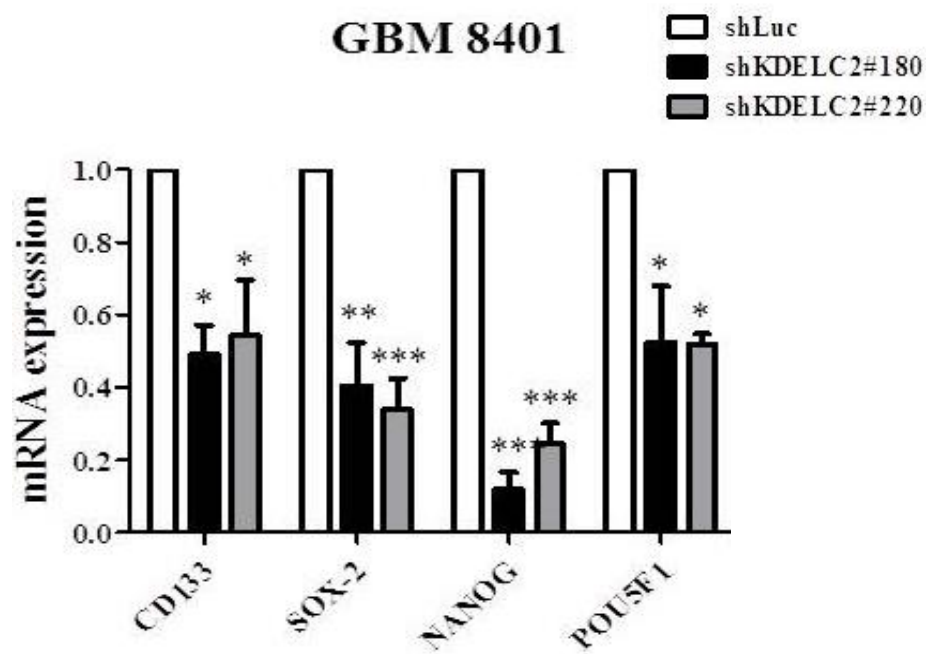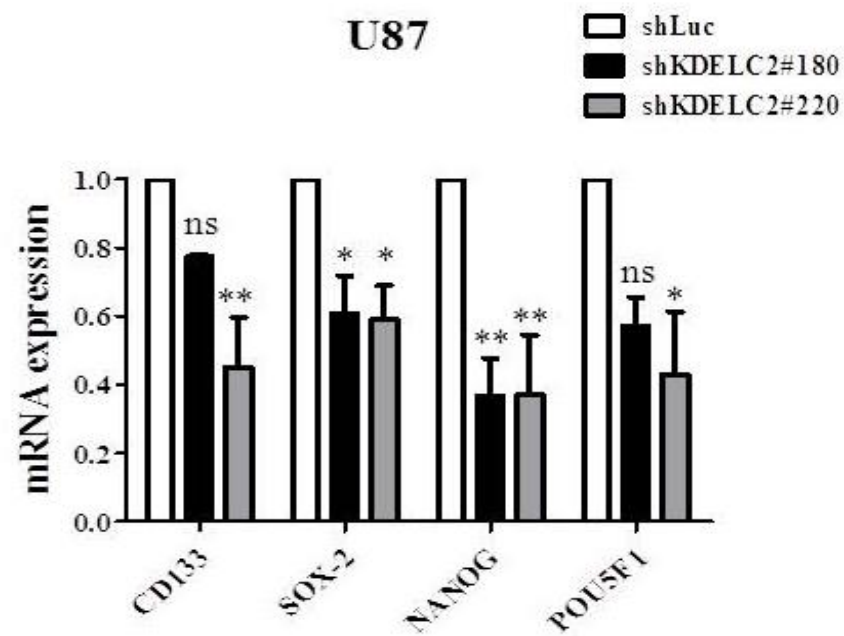

Supplement: Supplementary file 1 [file biomedicines-08-00339-s001.zip › biomedicines-910361-supplementary final/Fig. S5.pdf]

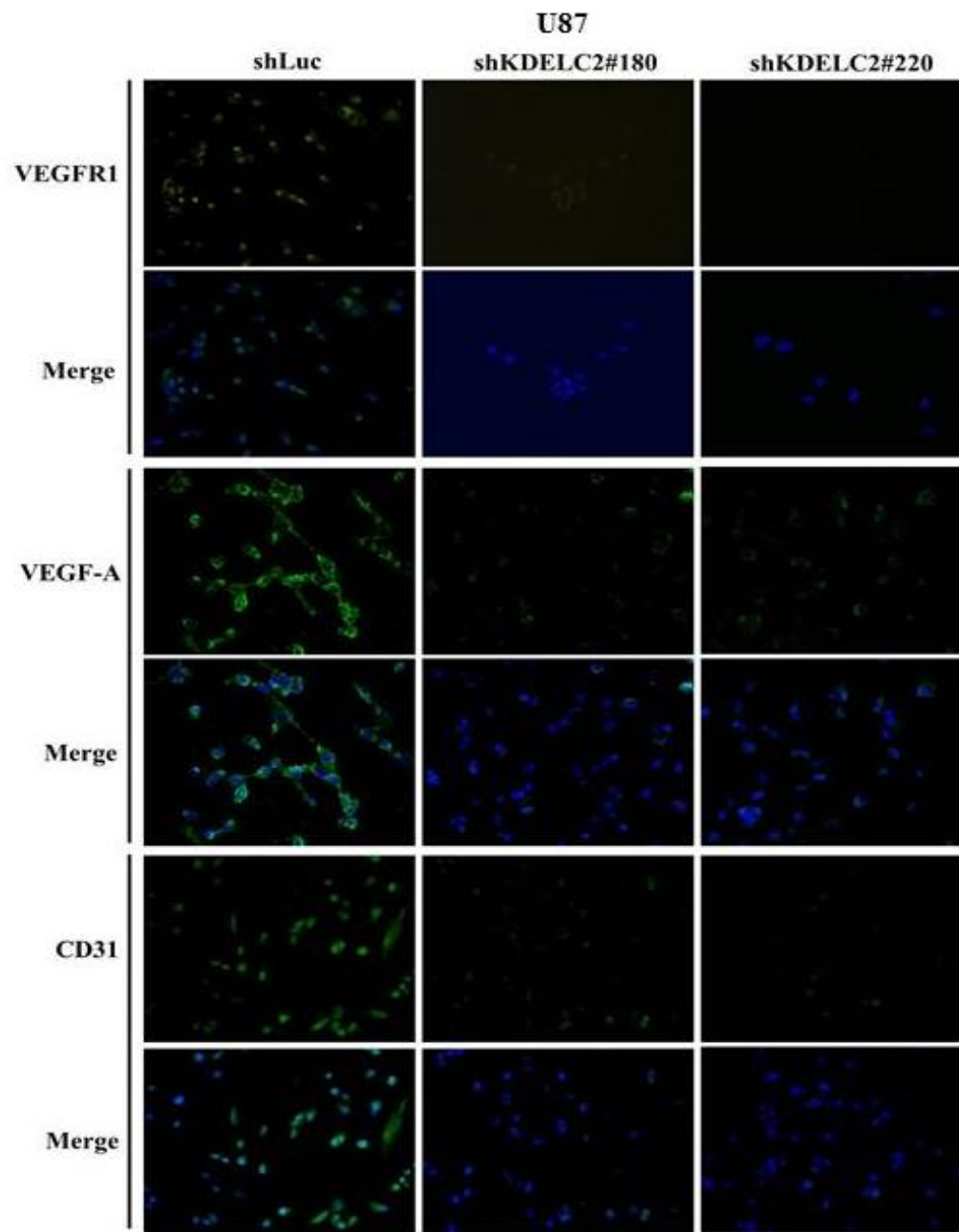

Supplement: Supplementary file 1 [file biomedicines-08-00339-s001.zip › biomedicines-910361-supplementary final/Fig. S6.pdf]
